# Validity and reproducibility of the ARL‐RSI score to assess psychological readiness before returning to sport after lateral ankle sprain

**DOI:** 10.1002/jeo2.12073

**Published:** 2024-07-02

**Authors:** Brice Picot, Olivier Grimaud, Gauthier Rauline, Ibrahim Haidar, Mohamad K. Moussa, Alexandre Hardy

**Affiliations:** ^1^ Interuniversity Laboratory of Human Movement Sciences Savoie MontBlanc University Chambéry France; ^2^ French Society of Sports Physical Therapist (SFMKS Lab) Pierrefitte‐surSeine France; ^3^ Department of Sports Surgery Clinique du Sport Paris France; ^4^ Department of Orthopedic Surgery Emirates Hospital Dubai United Arab Emirates; ^5^ Department of Orthopedic Surgery Group Hospitalier Sélestat‐Obernai Sélestat France

**Keywords:** ALR‐RSI, ankle sprain, return to sport

## Abstract

**Purpose:**

Although multiple scales exist to evaluate psychological readiness before returning to sport post‐lateral ankle sprain (LAS), no score has been validated specifically for LAS or chronic ankle instability. The main aim of the study is to evaluate the validity and reproducibility of the ankle ligament reconstruction‐return to sport injury (ALR‐RSI) scale in assessing psychological readiness after LAS and its ability to identify patients who can return to their preinjury level.

**Methods:**

A total of 64 patients (35 females and 29 males; 33.8 ± 13.2 years) who recently experienced an acute LAS were included in this study. All patients participated in a predictive validation component of the study and were assessed at 2 and 4 months following an LAS. The ALR‐RSI was completed twice by 20 patients at a 7‐day interval to evaluate the reliability of the score in patients suffering from LAS.

**Results:**

The ALR‐RSI was significantly (*p* < 0.001) and positively correlated with the other scores. The correlation was strong with the Foot and Ankle Ability Measure Sports: *r* = 0.77; 95% confidence interval [CI]: 0.78–0.83) and moderate with the American Orthopaedic Foot and Ankle Society (*r* = 0.69; 95% CI: 0.60–0.78) and the Foot and Ankle Ability Measure Activities of Daily Living Scores (*r* = 0.63; 95% CI: 0.51–0.72). Two‐month ALR‐RSI scores had good ability for predicting nonreturners at 4 months (area under the curve = 0.76; 95% CI: 0.6–0.9; *p* = 0.005). A Youden index of 0.51 was observed at an ALR‐RSI score of 46%, corresponding to a sensitivity of 67% and specificity of 83%. Test–retest reliability of the ALR‐RSI was excellent, with an ICC of 0.98 (95% CI: 0.96–0.99), a standard error of measurement of 3.02% and a minimum detectable change of 8.37%.

**Conclusions:**

The results of the current study validated the ALR‐RSI as an important questionnaire to assess psychological readiness to return to sport after LAS.

**Level of Evidence:**

Level II prospective cohort study.

**Abbreviations**
ALR‐RSI: ankle ligament reconstruction‐return to sport injury
AOFAS: American Orthopaedic Foot and Ankle Society
AUC: area under the curve
CAI: chronic ankle instability
CI: confidence interval
FAAM: foot and ankle ability measure
FAAMadl: Foot and Ankle Ability Measure Activities of Daily Living
FAAMsport: Foot and Ankle Ability Measure Sports
ICC: intraclass correlation coefficient
LAS: lateral ankle sprain
MDC: minimum detectable change
ROC: receiver operating characteristic
RTS: return to sport
SEM: standard error of measurement

## INTRODUCTION

Lateral ankle sprain (LAS) is one of the most common musculoskeletal injuries among athletes [17]. Its incidence ranges from 0.75 to 0.89 per 1000 in athletes participating across 15 sports in the National Collegiate Athletic Association from the 1988–1989 to the 2003–2004 seasons [8]. The rate of ankle sprain in professional football players ranges from 10% to 15% of all injuries [8], although the actual prevalence might be higher as over 50% of those with LAS do not seek medical attention [7].

Ankle sprains can lead to various degrees of functional impairments, such as decreased performance, absence from competition and psychological effects [23]. More than 20% of patients suffer from chronic symptoms, such as recurrent sprains, leading to chronic ankle instability (CAI) [20]. Despite advancements in surgical techniques to address this instability—from ligament repair to ligament reconstruction—conservative treatment followed by functional rehabilitation remains the primary approach for managing LASs, yielding satisfactory results [9, 16].

Returning to the previous level of activity or sport is a major concern for both patients and physicians [5]. However, determining an accurate timeline for returning to sport is challenging due to factors, such as residual pain or instability, which are influenced by the severity of the sprain, inadequate rehabilitation or even premature return to sport (RTS) [2, 5, 11, 13]. Even though physical impairment is a major hurdle to a successful RTS, psychological factors also play an important role in RTS [3, 15].

Recent consensus on RTS recommends objectively assessing the psychological state of the patient before making a decision [19]. Although multiple scales exist to evaluate psychological readiness before returning to sport, none of them are specific to LAS.

The ankle ligament reconstruction‐return to sport injury (ALR‐RSI) scale was developed by Sigonney et al. as a valid tool to assess psychological readiness after lateral ankle ligament reconstruction [18]. Similar psychological scores have been validated for ACL reconstruction, shoulder instability and femoroacetabular impingement syndrome [6, 21, 22]. However, despite the frequent incidence of LAS, no scale has yet been validated to assess psychological readiness for RTS after an ankle sprain or CAI.

The main purpose of this study was to evaluate the validity and reproducibility of the ALR‐RSI in a population that suffered LASs and to assess the ability of the ALR‐RSI to correctly identify patients who can return to their preinjury level. The hypothesis is that the ALR‐RSI can be a valid tool for predicting RTS in patients suffering from LASs.

## MATERIALS AND METHODS

### Participants

A total of 64 patients (35 females and 29 males; average age 33.8 ± 13.2 years) who recently experienced an acute LAS were included in this study based on the following criteria:

- Above 18 years old.
- A medically diagnosed episode of LAS that occurred within the past 3 months.
- Regular practice of at least one sport.

The diagnosis of the ankle sprain was confirmed during a consultation with a single orthopaedic surgeon. Patients who had discontinued their sports activities before the sprain episode were not included in the study. The severity stage of the sprain was not considered. Patients were included from May 2021 to December 2021. Participants also took part in a predictive validation component of the study and were assessed at 2 and 4 months following the LAS.

### Measures

#### ALR‐RSI scale

This 12‐item scale is designed to measure psychological readiness for RTS after ankle sprains or reconstruction surgery [14, 18]. It contains 12 items, thought to capture the psychological readiness before RTS, and includes three domains: emotions (five items), confidence in performance (five items) and risk evaluation (two items). The total score was equal to the sum of the 12 answers and divided by 1.2 to obtain a percentage. Each item goes from 0 (lowest psychological readiness) to 100 (highest psychological readiness). This scale was previously validated in patients undergoing ankle ligament reconstruction or modified Broström–Gould procedure [14, 18]. A French version of the scale has also been recently validated [1].

The patient‐reported outcome measures used were the American Orthopaedic Foot and Ankle Society (AOFAS) scale [10] and the two subscales (Activities of Daily Living and Sports) of the Foot and Ankle Ability Measure Score (FAAMadl and FAAMsport) [12].

After giving their consent, patients completed a self‐administered questionnaire, which included the ALR‐RSI scale, the AOFAS score, the FAAM score and several questions regarding their RTS. In addition, the ALR‐RSI was completed twice by 20 patients at a 7‐day interval to evaluate the reliability of the score in patients suffering from LAS.

### Statistical analysis

All participants completed the 12‐item ALR‐RSI at 2 or 4 months after LAS and answered a question that specifically asked whether they had returned to their preinjury level of sport. The response options were ‘no’, ‘yes, but at a lower level’ or ‘yes, at the same or higher level’. Based on the participant responses, the validity of the construct was tested between the ALR‐RSI and the AOFAS and FAAM scores (both subscales) by Pearson's correlation coefficient. The degree of correlation (*r*) was defined according to the following guidelines: ≤0.2 = very weak, >0.2–0.4 = weak, >0.4–0.7 = moderate, >0.7–0.9 = strong and >0.9 = very strong.

To evaluate the ability of the test to distinguish patients at 2 and 4 months following injuries, discriminant validity was tested using a paired sample *t* test (Student) among patients in the predictive validation component sample. Moreover, discriminant validity was also tested at 4 months between the group of patients who ‘had returned to do sport’ and the group that ‘had not returned to do sport’ by the Mann−Whitney test.

The predictive validity of the ALR‐RSI was assessed by using receiver operating characteristic (ROC) curve statistics. The largest Youden index value (sensitivity+ specificity−1) was used to determine the ideal cut‐off score using the 2‐month ALR‐RSI score and 4‐month return‐to‐sport status of the patients in the predictive validation group. Two predictive analyses were conducted. First, the predictive ability for returning to the same or higher preinjury level of sport was determined. For this analysis, RTS was dichotomised as patients who had returned to their preinjury level of sport or a higher level versus those who had not (no sport or lower level). Second, the predictive ability for not returning to sport was determined. For this analysis, RTS was dichotomised as patients who had not returned to any form of sport compared with those who had (lower than preinjury level or the same as preinjury level or higher). Similarly, ROC curve analysis was used to evaluate the ability of the four scales to correctly identify individuals who returned to sport at the same preinjury level by comparing the area under the curve (AUC) of each scale.

Test–retest reliability of the ALR‐RSI was analysed using intraclass correlation coefficient (ICC, two‐way mixed‐effects model for agreement) and interpreted as ‘poor’ for values below 0.5, ‘moderate’ between 0.5 and 0.75, ‘good’ between 0.75 and 0.9 and ‘excellent’ above 0.9 [10]. Absolute reliability was quantified by the standard error of measurement (SEM) from the equation: SEM = SD × √1 – ICC and interpreted using the minimum detectable change (MDC) using the equation:

MDC=1.96×√2×SEM.

This value is defined as the smallest change in score that passes the threshold of error for the scale. For all parameters, normality was assessed using the Shapiro–Wilk test. All statistical analyses described above were performed with SPSS software version 22 (SPSS Inc.), and the *α* level of significance was fixed at *p* < 0.05. Effect sizes (Cohen's *d*) were also reported with 0.2, 0.5 and 0.8 representing small, moderate and large effects, respectively.

## RESULTS

### Validity

The ALR‐RSI was significantly (*p* < 0.001) and positively correlated with the other scores. The correlation was strong with the FAAMsport: *r* = 0.77; 95% confidence interval [CI]: 0.78–0.83) and moderate with the AOFAS (*r* = 0.69; 95% CI: 0.60–0.78) and the FAAMadl scores (*r* = 0.63; 95% CI: 0.51–0.72).

### Discriminant validity

A significant difference (*p* < 0.001) was found in the ALR‐RSI, AOFAS and FAAMsport between patients at 2 and 4 months, but a very large effect was only observed for the ALR‐RSI score. Conversely, no significant difference was observed between the two periods based on the FAAMadl (Table 1).

**Table 1:** Means (SD) scores in percentage at 2 and 4 months of the ALR‐RSI, AOFAS, FAAMadl and FAAMsport.

Measure	*n* = 64	2 months	4 months	*p* Value	Cohen's *d*
ALR‐RSI		45 (±21.2)	83.3 (±21.2)	<0.001	−1.3
AOFAS		70.5 (±16.7)	68.3 (±21.2)	<0.001	−0.75
FAAMadl		85.1 (±14.4)	93.5 (±7.5)	<0.001	−0.61
FAAMsport		59.7 (±22.3)	80.1 (±17.7)	<0.001	−0.96

Abbreviations: ALR‐RSI, ankle ligament reconstruction‐return to sport injury; AOFAS, American Orthopaedic Foot and Ankle Society; FAAMadl, Foot and Ankle Ability Measure Activities of Daily Living; FAAMsport, Foot and Ankle Ability Measure Sports; SD, standard deviation.

At 2 months, no participants returned to sport at the same preinjury level. A highly significant difference, with a strong effect size (*p* < 0.001; *d* = −1.125) was found in the ALR‐RSI between the subgroup of 15 patients who returned to sport, regardless of the level of play and the 39 who did not: 60.3 (±13.8%) versus 40.3 (±21%), respectively. AOFAS and FAAM scores were also able to discriminate those two groups (Table 2) but with moderate effect size.

**Table 2:** Means (SD) scores in percentage at 2 months between patients who returned to sport (regardless of the level of play) and those who did not.

Measure	Return to sport (*n* = 15)	Not returned to sport (*n* = 49)	*p* Value	Cohen's *d*
ALR‐RSI	60.3 (±13.8)	40.3 (±21)	<0.001	−1.019
AOFAS	80.6 (±10.5)	67.4 (±17.1)	0.007	−0.828
FAAMadl	92.1 (±5.4)	82.9 (±15.6)	0.03	−0.656
FAAMsport	73.8 (±13.1)	55.4 (±22.9)	0.004	−0.871

Abbreviations: ALR‐RSI, ankle ligament reconstruction‐return to sport injury; AOFAS, American Orthopaedic Foot and Ankle Society; FAAMadl, Foot and Ankle Ability Measure Activities of Daily Living; FAAMsport, Foot and Ankle Ability Measure Sports; SD, standard deviation.

At 4 months, a highly significant difference with strong effect size (*p* < 0.001; *d* = −1.338) was found in the ALR‐RSI between the subgroup of 32 patients, who returned to playing sport at the same level of preinjury and the 20 who did not (inferior level): 80.9 (±15.5) versus 58.8 (±18.1), respectively. AOFAS and FAAM scores were also able to discriminate those two groups with strong effect sizes (Table 3).

**Table 3:** Means (SD) scores in percentage at 4 months between patients who returned to the same level of sport and those who did not.

Measure	Return to sport at the same level (*n* = 32)	Not returned to sport at the same level (*n* = 20)	*p* Value	Cohen's *d*
ALR‐RSI	80.9 (±15.5)	58.8 (±18.1)	<0.001	−1.338
AOFAS	89.7 (±10.5)	77.3 (±8.5)	<0.001	−1.263
FAAMadl	96.8 (±4.4)	92.4 (±5.9)	0.004	−0.869
FAAMsport	89.3 (±10.4)	75.9 (±17)	<0.001	−1.001

Abbreviations: ALR‐RSI, ankle ligament reconstruction‐return to sport injury; AOFAS, American Orthopaedic Foot and Ankle Society; FAAMadl, Foot and Ankle Ability Measure Activities of Daily Living; FAAMsport, Foot and Ankle Ability Measure Sports; SD, standard deviation.

### Predictive ability for returning to preinjury level of sport or higher at 4 months

**Figure 1:**
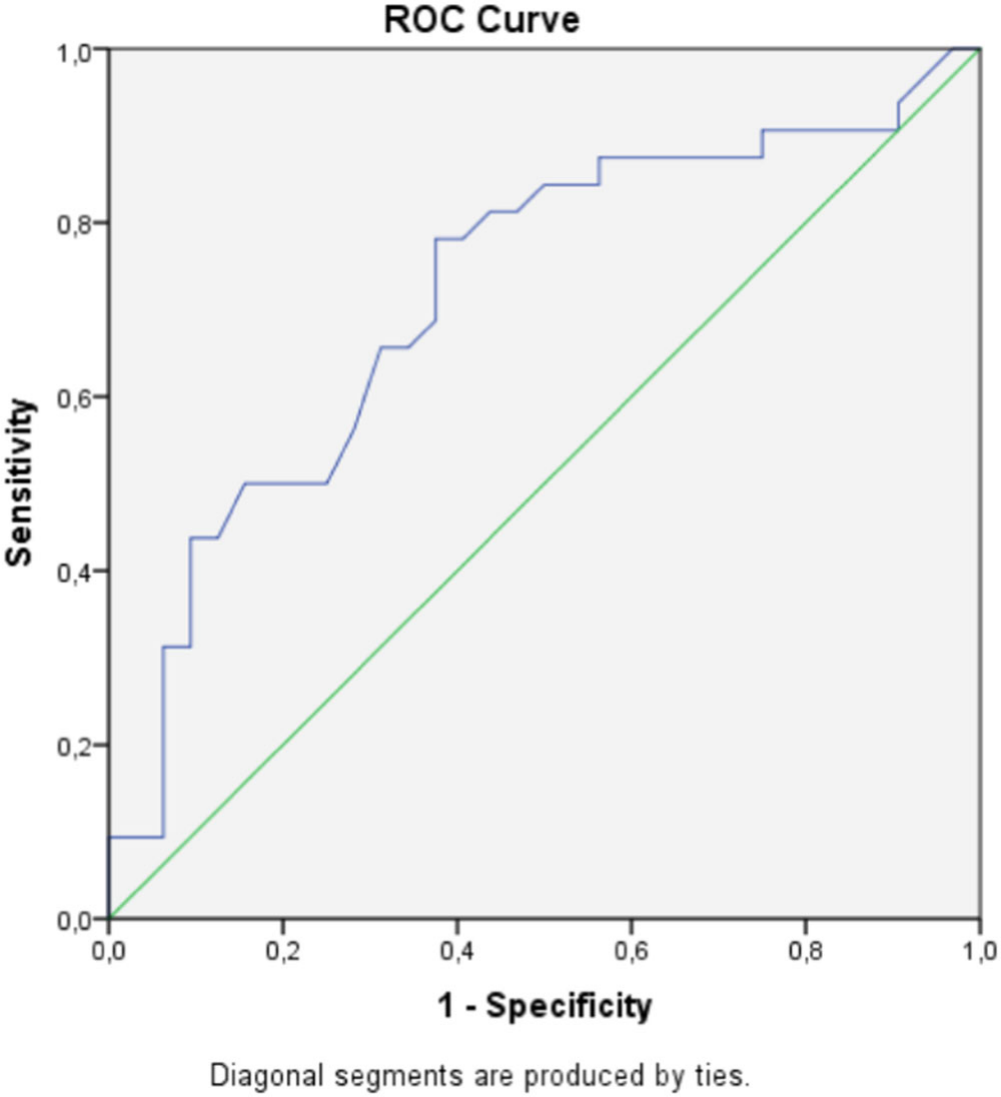
Receiver operating characteristic (ROC) curve for ALR‐RSI scale for predicting a return to preinjury level of sport or higher.

Of the 64 patients in the predictive validation component, only 32 (50%) returned to their preinjury level of sport at 4 months. Two‐month ALR‐RSI scores had a fair predictive ability to identify those patients (AUC = 0.72; 95% CI: 0.60–0.85; *p* = 0.03) (Figure 1). A Youden index of 0.41 was observed at an ALR‐RSI score of 46%, corresponding to a sensitivity of 78% and specificity of 63%.

### Predictive ability for not returning to sport

**Figure 2:**
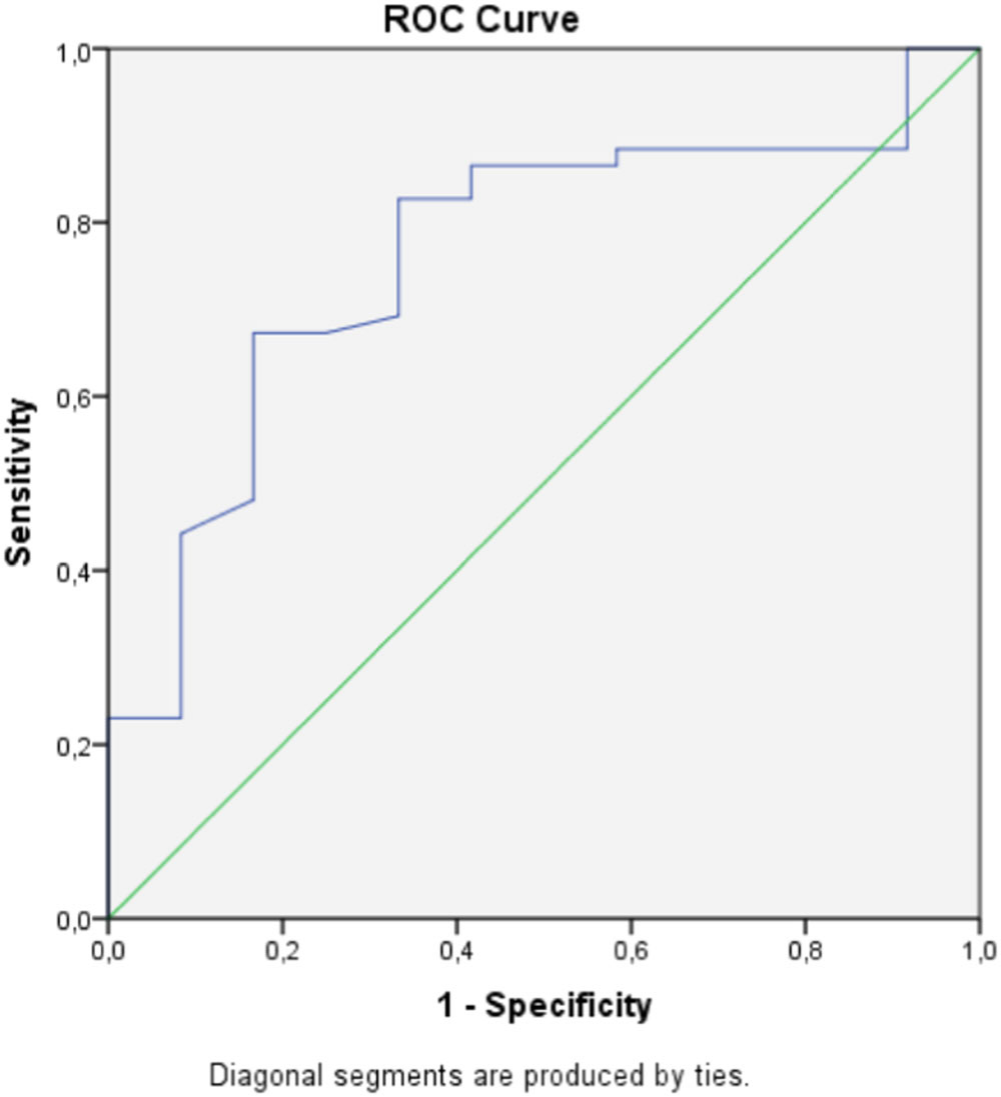
Receiver operating characteristic (ROC) curve for ALR‐RSI scale for predicting not returning to sport.

Of the 64 patients, 12 (18.8%) had not attempted any sport at 4 months. Two‐month ALR‐RSI scores had good ability for predicting nonreturners at 4 months (AUC = 0.76; 95% CI: 0.6–0.9; *p* = 0.005) (Figure 2). A Youden index of 0.51 was observed at an ALR‐RSI score of 46%, corresponding to a sensitivity of 67% and specificity of 83%.

### Reliability

Test–retest reliability of the ALR‐RSI was excellent, with an ICC of 0.98 (95% CI: 0.96–0.99), an SEM of 3.02% and an MDC of 8.37%.

## DISCUSSION

The main finding of this study was that the ALR‐RSI is a valid and reproducible scale for the evaluation of psychological readiness before RTS after an LAS in an active population.

Valid and reliable scales are crucial for assessing the complexity and severity of a functional problem in patients, providing a common platform for surgeons and physicians and allowing for the comparison of results from different treatments. The ARL‐RSI scale was developed to assess the psychological status of patients after undergoing ankle ligament reconstruction [18] and was recently validated for patients undergoing the modified Broström–Gould procedure for chronic CAI [14]. The results of this study revealed a significant correlation between the ARL‐RSI score and another questionnaire used to determine the psychological readiness of subjects to RTS.

These findings confirm the importance of psychological factors in RTS after an LAS. The ALR‐RSI, in combination with functional tests, could assist clinicians in making RTS decisions [15, 19]. Numerous studies have highlighted the significance of psychological factors as key indicators for a successful return. Many experts have recommended assessing the psychological status of patients to determine the appropriate timeline for RTS [15, 19]. Clanton et al. emphasised the importance of subjective data for determining the capability of RTS [4]. Decisions about RTS that are based solely on functional and biological outcomes should be dropped in favour of a multidisciplinary framework relying on functional and psychological factors. The ALR‐RSI facilitates the assessment of psychological readiness related to RTS, enabling clinicians to gauge patient confidence and assess the risk of recurrent sprain. In the context of anterior cruciate ligament injury, the ACL‐RSI scale has become an integral part of the rehabilitation protocol since a strong correlation has been demonstrated between the scale and RTS in patients undergoing ACL reconstruction.

There are several limitations to the study. First, there may be a selection bias due to the retrospective nature of the study; second, the ALR‐RSI was not originally developed for LAS, although it has recently been validated for ankle instability following anatomic ankle reconstruction and the modified Broström–Gould procedure.

## CONCLUSION

The results of the current study validated the ALR‐RSI as an important questionnaire to assess psychological readiness for RTS after LAS. The ALR‐RSI has to be considered a crucial RTS tool since it could help practitioners during the rehabilitation process to identify patients who will be able to RTS.

## AUTHOR CONTRIBUTIONS

**Brice Picot**: Conceptualisation; methodology; data analysis; writing—original draft; supervision. **Olivier Grimaud**: Investigation; data collection; methodology; writing—review and editing. **Gauthier Rauline**: Data curation; data collection; formal analysis; writing—review and editing. **Ibrahim Haidar**: Investigation; data collection; resources, writing—review and editing. **Mohamad K. Moussa**: Conceptualisation; methodology; writing—review and editing. **Alexandre Hardy**: Project administration; supervision; visualisation; writing—review and editing.

## CONFLICT OF INTEREST STATEMENT

A. H.: Consultant for Arthrex and Depuy. The remaining authors declare no conflict of interest.

## ETHICS STATEMENT

This study involves human participants and was approved by the ethics committee. Patients and/or the public were not involved in the design, conduct, reporting or dissemination plans of this research. Feedback on the collection of information for the database is encouraged.

## Data Availability

Data are available upon reasonable request.
